# Benchmark CCSD(T) and Density Functional Theory Calculations of Biologically Relevant Catecholic Systems

**DOI:** 10.1021/acs.jpcb.4c08356

**Published:** 2025-05-14

**Authors:** Joshua Harle, Mauricio Cafiero

**Affiliations:** † School of Chemistry, 1724 University of Birmingham, Birmingham B15 2TT, U.K.; ‡ School of Chemistry Food and Pharmacy, 6816 University of Reading, Reading RG6 6AH, U.K.

## Abstract

Approximate complete basis set CCSD­(T), MP2, and HF calculations are performed for thirty-two catechol-containing complexes. These complexes, which include metal-coordination, hydrogen-bonding, π-stacking, and other, weaker interactions, are representative of the types of noncovalent interactions that catechols undergo when binding to proteins in the body, such as in the biosynthesis of dopamine. The catechols studied include the neutral catechol and dinitrocatechol molecules, as well as the charged dopamine and DOPAC molecules. Calculations with twenty-one density functional theory methods with triple and quadruple-ζ basis sets are evaluated against the CCSD­(T) benchmarks to ascertain their accuracy. It is found that MN15, M06-2X-D3, ωB97XD, ωB97M-V, and CAM-B3LYP-D3 provide good accuracy when compared with CCSD­(T)/CBS calculations for these systems and may be used for the study of relevant biological systems. The local DPLNO CCSD­(T) method is also evaluated against the CCSD­(T)/CBS energies for a subset of the complexes and found to agree within 1–3%, with a maximum difference of 0.26 kcal/mol.

## Graphical abstract

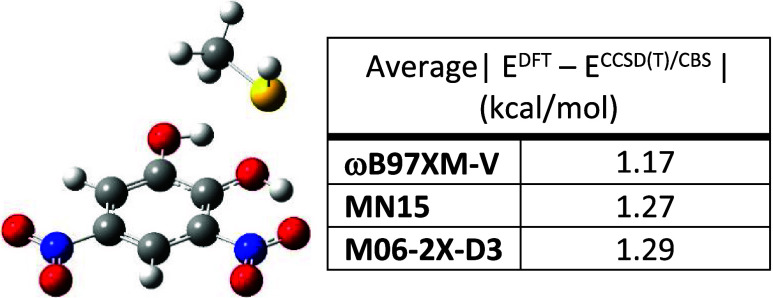

## 1 Introduction

Parkinson’s Disease (PD) is the second most common age-related neurodegenerative disease—after Alzheimer’s Disease—with the total number of cases reported in 2016 rising to 6.1 million, and with the predicted number of PD cases increasing by 65% between 2005 and 2030 globally.^1,2^ Though the cause of PD is unknown, and thus it is currently incurable, it is widely accepted that the cause of the tremors experienced by those suffering from PD is due to the degradation of dopaminergic neurons in the substantia nigra, leading to decreased dopamine production within the brain.^3^ Dopamine levels can be increased by administration of L-DOPA, which crosses the blood-brain barrier and is converted into dopamine.^4^

There are eight enzymes that are involved in dopamine and L-DOPA synthesis and metabolism: phenylalanine hydroxylase, tyrosine hydroxylase, and DOPA decarboxylase (synthesis) and catechol-*o*-methyltransferase (COMT), monoamine oxidase (MAOB), aldehyde dehydrogenase, tyrosinase, and sulfotransferase (SULT, metabolism). When designing a drug for PD, the first three enzymes should not be inhibited (to preserve the body’s natural dopamine production) while the last five can be targeted for inhibition (to maintain high dopamine levels).^4^ Ligands in these eight enzymes are held in place largely by four types of forces: ionic/primarily electrostatic, hydrogen bonds/primarily dipole–dipole, π-stacking/primarily induction and dispersion, and other weak forces (weaker dipole/dipole and induction and dispersion). Examining the forces experienced by dopamine in all of the eight enzymes noted above as an example,^5^ we can categorize the forces as 17.3% ionic, 11.8% hydrogen bonds, 24.4% π-stacking or other ring/ring interactions, and 46.5% other weak interactions. Thus, in the process of computational drug design for PD, all of these forces must be well-described by the method employed. In this work, benchmark CCSD­(T) structures and interaction energies for thirty-two catecholic complexes, which are directly relevant to Parkinson’s disease drug design, will be established, and twenty-one Density Functional Theory (DFT) methods will be evaluated against these interactions.

When applying *ab initio* methods to computational drug design, DFT is often chosen as it offers a good balance of accuracy and speed of calculation. New functionals are typically benchmarked or calibrated against databases that include nonbonded interactions such as those discussed above. In many cases of database calibration/testing, the benchmark calculations are performed on a specified structure, such as in the case of the database developed by Jurečka et al.,^6^ which is commonly used to benchmark DFT methods. This database has experimental geometries, or geometries optimized with high-level *ab initio* methods and a large basis set, which are used for interaction energy calculations. The work of Zhao and Truhlar^7^ shows the importance of geometry optimizations when testing DFT methods against benchmark standards for biological systems. In their work, they note that not all DFT methods tested against databases can find the minima of biologically relevant complexes and that the performance of M052X for binding energies is improved when an optimization is done with the same method. Thus, calibration against a known geometry may obscure the fact that a given method is qualitatively incorrect, or portray it as less accurate than it is. DFT-based geometry optimizations for eight of the 30 two complexes studied here will thus be performed to assess how much of a difference a separate optimization would make on interaction energies.

Accuracy of each DFT method will be evaluated by comparison against a Coupled Cluster Singles and Doubles and perturbative triples [CCSD­(T)] calculation^8^ for each type of interaction (see above). Average absolute difference (AAD) errors for each type of interaction for each DFT method studied will be presented:

1 $${\mathrm{AAD}}_{\mathrm{DFT}/\mathrm{type}}=\sum_{i}\left|E_{i}^{\mathrm{DFT}/\mathrm{type}}-E_{i}^{\mathrm{CCSD}\left(T\right)/\mathrm{type}}\right|$$

Where *E* is the interaction energy calculated for a specific DFT method or with CCSD­(T) and the index *i* runs over the complexes of each type (*i.e*. metal/ionic, h-bonds, *etc*). So, for example, the average absolute difference for ionic interactions using the B3LYP method would be *AAD*_*B3LYP/ionic*_ and *i* would run over all ionic complexes. We will also report the total average absolute error across all interaction types:

2 $${\mathrm{AAD}}_{\mathrm{DFT}/\mathrm{total}}=\sum_{i=1}^{\mathrm{all}}\left|E_{i}^{\mathrm{DFT}}-E_{i}^{\mathrm{CCSD}\left(T\right)}\right|$$

### 1.1 DFT Methods Evaluated

The DFT functionals studied in this work were chosen to examine the effects of exact exchange (HF exchange) on the intermolecular interactions studied, both in the global hybrid and the range-separated hybrid forms. The effects of empirical dispersion terms were also examined.^9,10^

The first functional examined was the local density approximation (LDA) method, SVWN,^11,12^ which includes only the local electron density in the calculation of the exchange and correlation energies:

3 $$E_{\mathrm{LDA}}^{\mathrm{DFT}}=E_{X}^{\mathrm{DFT}}\left(\rho \right)+E_{C}^{\mathrm{DFT}}\left(\rho \right)$$

The LDA method is included in this study for comparison only, although, by fortuitous chance, it does predict accurate interaction energies for π-stacking and the other weak interactions studied here. In order to set a baseline for the various hybrid functionals studied, several GGA methods are examined. These functionals, in simple terms, have two terms for the DFT exchange and correlation energies, each of which contains both the electron density and the gradient of the density:^13^

4 $$E_{\mathrm{GGA}}^{\mathrm{DFT}}=E_{X}^{\mathrm{DFT}}\left(\rho ,\nabla p\right)+E_{C}^{\mathrm{DFT}}\left(\rho ,\nabla p\right)$$

Another baseline for comparison for the hybrid methods can be had from examining meta-GGA methods, which include the kinetic energy density, τ (or the Laplacian of the electron density), as well as the electron density and its gradient:^13^

5 $$E_{\mathrm{MGGA}}^{\mathrm{DFT}}=E_{X}^{\mathrm{DFT}}\left(\rho ,\nabla p,\tau \right)+E_{C}^{\mathrm{DFT}}\left(\rho ,\nabla p,\tau \right)$$

The simplest type of hybrid functionals examined here are the global hybrids, which include a percentage (X) of exact, or HF exchange:

6 $$E_{\mathrm{Hybrid}}^{\mathrm{DFT}}={\left(1-X\right)E}_{X}^{\mathrm{DFT}}+{\mathrm{XE}}_{X}^{\mathrm{HF}}+E_{C}^{\mathrm{DFT}}$$

The range-separated hybrid functionals include a portion of the HF exchange energy. In this case, though, the amount of HF exchange is not static, but varies based on the system. In general, the Coulomb operator is separated into a short-range (SR) and long-range (LR) term, based on a scaling factor ω:

7 $$\frac{1}{r}=\frac{1-\mathrm{erf}\left(\omega r\right)}{r}+\frac{\mathrm{erf}\left(\omega r\right)}{r}$$

which results in the total DFT energies being divided into SR and LR terms:

8 $$E_{\mathrm{RS}}^{\mathrm{DFT}}=E_{X,\mathrm{SR}}^{\mathrm{DFT}}+E_{X,\mathrm{LR}}^{\mathrm{DFT}}+E_{C}^{\mathrm{DFT}}$$

Different range-separated methods differ in whether HF exchange is applied to the SR portion, the LR portion, or both. Finally, the empirical-dispersion-corrected functionals include any of the above, along with the addition of a parametrized dispersion energy term:

9 $$E_{D}^{\mathrm{DFT}}=E_{X}^{\mathrm{DFT}}+E_{C}^{\mathrm{DFT}}+E_{D}$$

The dispersion term has several forms in use, including the D2,^9^ D3,^10^ and D3BJ^9^ forms.

In order to examine how the inclusion of exact exchange, kinetic energy density, and dispersion affects the ability of a DFT functional to accurately model the CCSD­(T)/CBS energies, several “families” of DFT methods have been studied. Each family progresses through one or more steps from GGA to Meta-GGA, to Hybrid (global and/or range-separated), and to empirical-dispersion-corrected. The first set studied was the dispersion-corrected GGA method, B97D3,^9^ a dispersion-corrected, range-separated hybrid, ωB97XD^14^ and a range-separated hybrid with nonlocal correlation, ωB97M-V.^15^ Next, a family of “Minnesota” functionals was studied, all of which are based on a meta-GGA starting point. M06L^16^ is a pure DFT method, and M06 ^17^ and M062X^17^ add 27 and 54% exact exchange, respectively. M062X-D3 adds empirical dispersion to M062X, MN12SX^18^ is a range-separated hybrid, and MN15 ^19^ is a later functional from the same authors. Next, a family based on the GGA BLYP^20,21^ functional is studied. This family includes BLYP, the global hybrid B3LYP,^22^ the range-separated hybrid CAM-B3LYP,^23^ and CAM-B3LYP-D3, which adds empirical dispersion to the previous functional. Next, the GGA PBE^24^ functional and two range-separated hybrid functionals derived from it, LC-ωHPBE,^25^ and ωPBEhPBE^26^ were studied. Finally, the GGA HCTH^27^ functional and two meta-GGA, hybrid functionals derived from it (τHCTHhyb,^28^ and BMK.^29^) were studied. One double-hybrid DFT method was tested, B2PLYPD3.^30^ Double-hybrids are largely avoided here as the time and compute needed for these calculations is larger than that needed by other DFT calculations and so are not as likely candidates for routine use.

## 2 Computational Methods

All calculations below were performed using the Gaussian 16 software,^31^ with the exception of the ωB97X-M calculations, which were run using Psi4 ^32^ and DLPNO-CCSD­(T) calculations, which were run using ORCA.^33^

### 2.1 Biologically Relevant Catecholic Systems

Thirty-two molecular complexes have been designed to mimic the types of interactions found between dopamine and the active sites of eight enzymes important in drug design for Parkinson’s Disease. Similarity of these complexes to the crystal structures of some of the eight enzymes mentioned above will be discussed below. These thirty-two complexes consist of four catecholic molecules (catechol, dinitrocatechol, dopamine, and DOPAC), each interacting with 8 counter-molecules. The first eight model complexes (ionic) are the four deprotonated catechols bound to a Mg^2+^ ion in an octahedral complex and a Zn^2+^ ion in an octahedral complex (see Figure 1). The deprotonated ligands carry a −1 charge (catechol and dinitrocatechol), a neutral charge (dopamine), and a −2 charge (DOPAC). These complexes are designed to mimic crucial interactions found between ligands and the active sites of catechol-*o*-methyltransferase and tyrosine hydroxylase. Ionic interactions are often the dominant interactions holding a ligand to an active site, as is the case with these two enzymes.^34,35^

**Figure 1:**
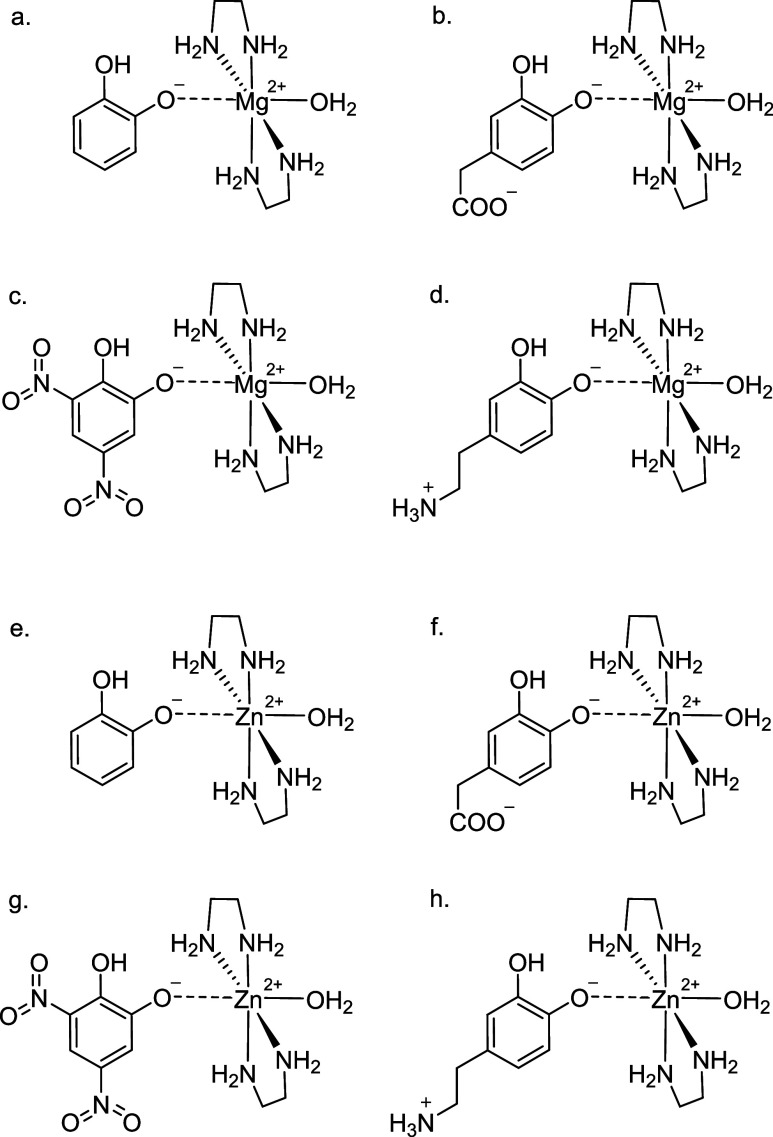
Metal complex model systems used in this study: Mg^2+^ in an octahedral complex with 2 ethylene diamine molecules, a water molecule, and (a) catechol, (b) DOPAC, (c) dinitro catechol and (d) dopamine, and Zn^2+^ in an octahedral complex with 2 ethylene diamine molecules, a water molecule, and (e) catechol, (f) DOPAC, (g) dinitro catechol and (h) dopamine.

The next eight complexes are models for hydrogen bonding. As stated above, 11.8% of the 127 total interactions between dopamine and the eight enzyme active sites are hydrogen bonds with interaction energies between ∼8 and ∼15 kcal/mol each, and so capturing these interactions is important for accurate overall modeling. These complexes (see Figure 2) consist of the four catechols hydrogen-bonded to methylamine and to methanol. The complexes with methylamine mimic interactions between the catechols and histidine, tryptophan, proline, glutamine, and asparagine residues in the enzyme active sites, while the complexes with methanol mimic interactions with serine, tyrosine, glutamine, and asparagine residues. The ligands are either neutral (catechol and dinitrocatechol), carry a +1 charge (dopamine), or carry a −1 charge (DOPAC).

**Figure 2:**
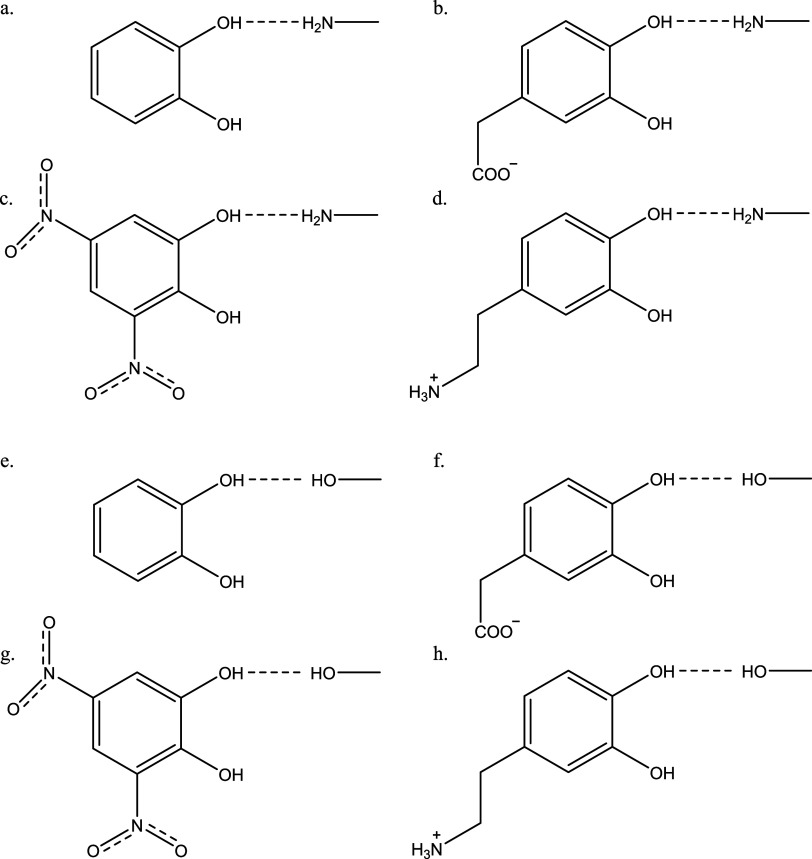
Hydrogen-bonded model systems used in this study: methyl amine hydrogen-bonded to (a) catechol, (b) DOPAC, (c) dinitro catechol and (d) dopamine, and methanol hydrogen-bonded to (e) catechol, (f) DOPAC, (g) dinitro catechol, and (h) dopamine.

The next eight complexes are models for π-stacking (see Figure 3). As stated above, π-stacking accounts for almost 25% of the interactions between dopamine and the active sites of the eight enzymes. The first four complexes are the four catechols stacked with benzene, to mimic the π-stacking with phenylalanine and tyrosine residues in the enzyme active sites, while the next four complexes are the four catechols stacked with indole, to mimic the π-stacking with tryptophan residues found in the enzyme active sites.

**Figure 3:**
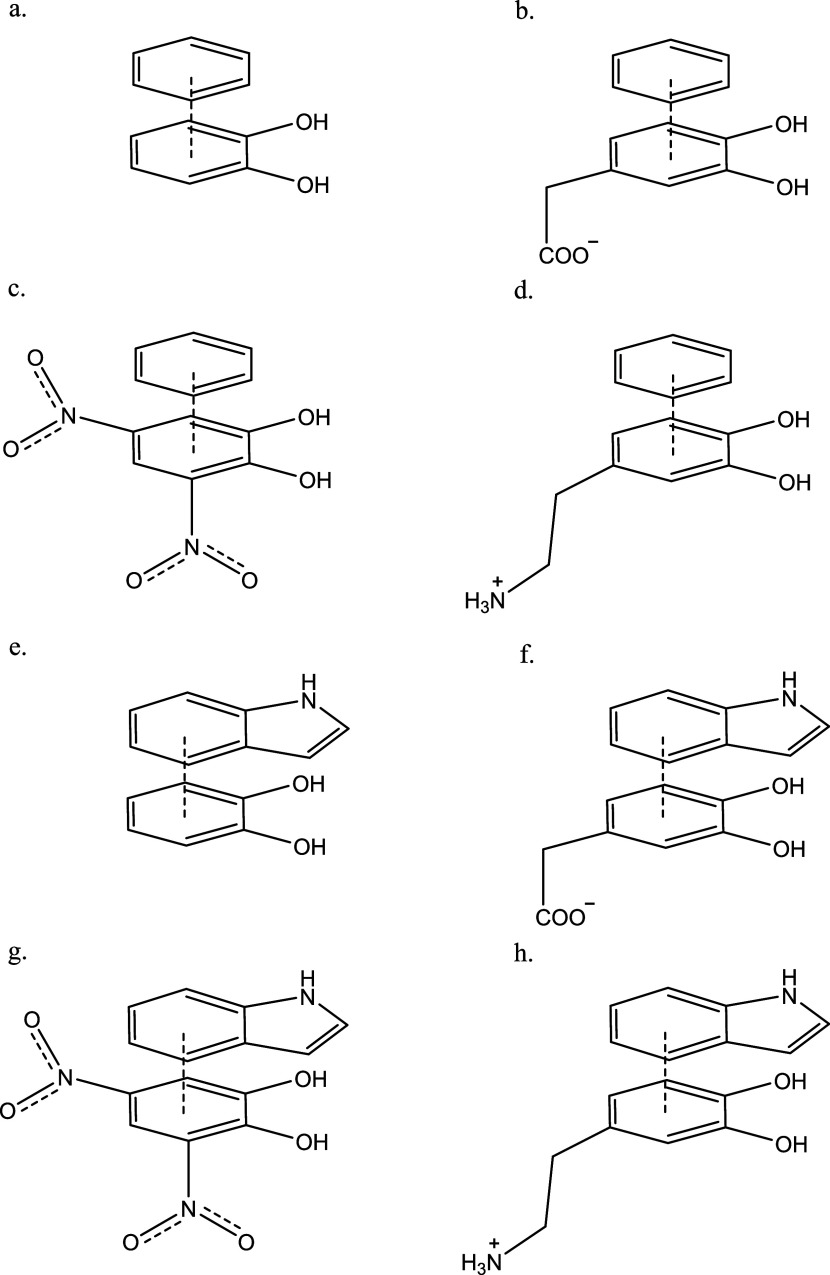
π-stacking systems used in this study: benzene stacked with (a) catechol, (b) DOPAC, (c) dinitro catechol, and (d) dopamine, and indole stacked with (e) catechol, (f) DOPAC, (g) dinitro catechol, and (h) dopamine.

The final eight complexes, shown in Figure 4, are models for “other” weak interactions and consist of the four catechols interacting with isobutane and with methanethiol. The complexes with isobutane mimic the interactions the ligands have with alanine, valine, leucine, and isoleucine residues in the enzyme active sites, while the complexes with methanethiol mimic interactions specifically with cysteine residues, and more broadly with any polar residues that do not form hydrogen bonds. These interactions account for almost 50% of the interactions between dopamine and the eight enzyme active sites, and so the accuracy of these complexes is crucial to the overall accuracy of the calculations.

**Figure 4:**
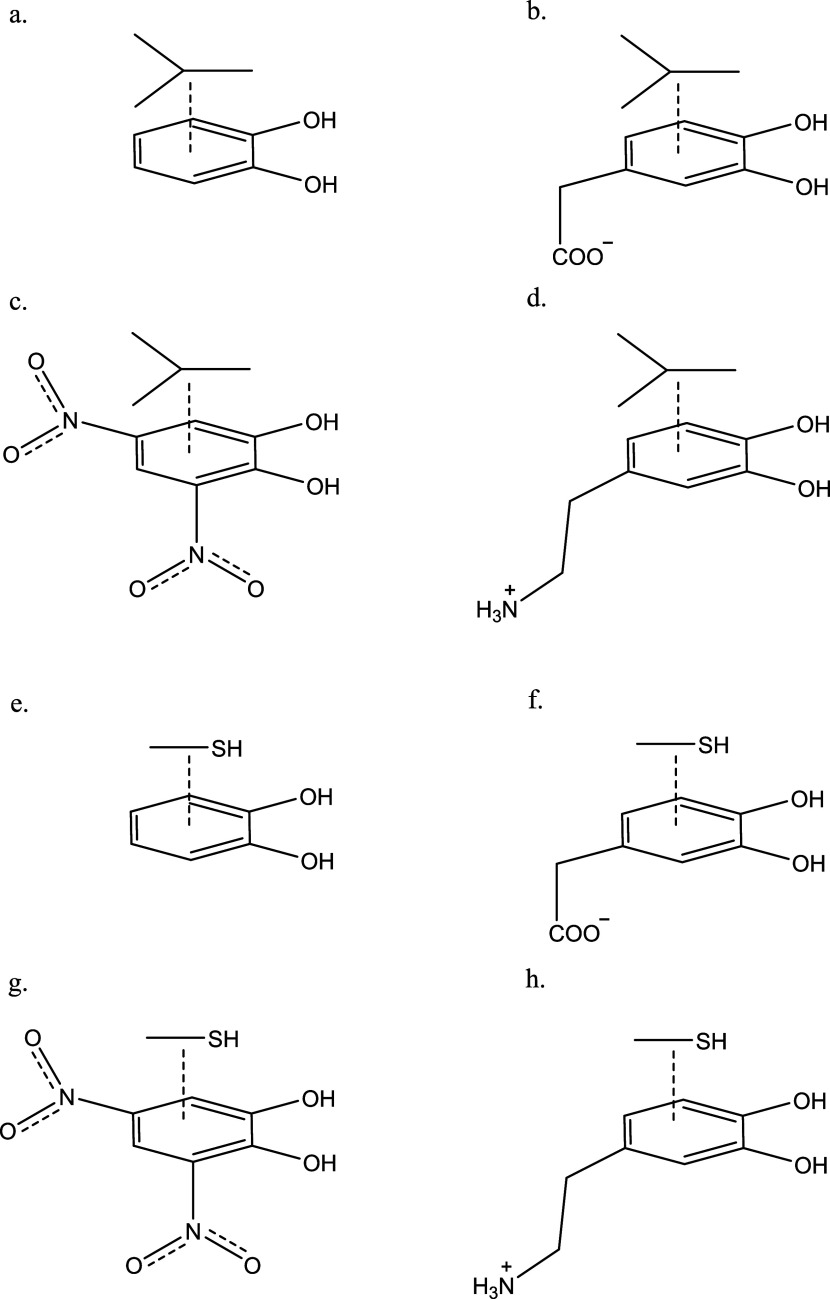
Other systems used in this study: isobutane complexed with (a) catechol, (b) DOPAC, (c) dinitro catechol, and (d) dopamine, and methanethiol complexed with (e) catechol, (f) DOPAC, (g) dinitro catechol, and (h) dopamine.

### 2.2 CCSD and MP2 Benchmark Calculations

The thirty-two complexes described above were preoptimized with M062*X*/6-31G, and then fully optimized using CCSD/cc-pVDZ or MP2/cc-pVDZ, as described in the results section. For complexes where dispersion interactions are prominent (such as in π-stacked complexes), these small basis set optimizations will yield longer intermolecular distances than in the CCSD­(T)/CBS limit. This is due to the underestimation of the dispersion forces with the smaller basis set. Work from Hickley and Rowley shows that CCSD with a cc-pVDZ basis set underestimates the experimental polarizability by about 22% and underestimates the CCSD/aug-cc-pVTZ polarizability by about 20% for a range of molecules.^36^ Since the induction and dispersion forces are proportional to the polarizability, it can be expected that the structures used here will have longer intermolecular distances than would be found experimentally, or by larger basis set CCSD or CCSD­(T) calculations. However, since the current work seeks to simply establish a baseline for comparisons, the small difference in structure should be acceptable.

Approximate complete basis set (CBS) CCSD­(T) energies for the complexes described above, as well as their individual components, were calculated according to the expression by used by Grimme and coauthors in several works,^37,38^ and benchmarked for hydrogen-bonded complexes by Jurecka and Hobza:^39^

10 $$E^{\mathrm{CCSD}\left(T\right)/\mathrm{CBS}}=E^{\mathrm{MP}2/\mathrm{CBS}}+\left(E_{\mathrm{corr}}^{\mathrm{CCSD}\left(T\right)/\mathrm{SB}}-E_{\mathrm{corr}}^{\mathrm{MP}2/\mathrm{SB}}\right)$$

where *E*_corr_ is the contribution to the total energy from correlation, and SB stands for a small basis set, which, in the current work, is cc-pVDZ. This basis set was used in the expression by Grimme and coauthors^37,38^ and was shown by Jurecka and Hobza to have good accuracy.^39^ Marshall et al. performed a study examining how the small basis set correction term in eq 10 behaves with different small basis sets for noncovalent interactions such as those studied here.^40^ While they showed that a double-ζ basis set has less accuracy for dipole-based interactions, its accuracy for dispersion and induction-based interactions is on-par with triple-ζ to sextuple-zeta basis sets. They further showed that the cc-pVDZ basis set used here has less than a 0.25 kcal/mol mean absolute deviation for combined hydrogen-bonded, dispersion-bonded, and mixed complexes in the S22 benchmark data set. Ehrlich et al. performed CCSD­(T)/CBS calculations and used the same small basis set for the correction term as was used here, and they estimate a maximum of 5% error for noncovalent complexes.^38^

The MP2 and HF CBS energies used in eq 10 and reported in Table 1 below were obtained with the formula by Halkier et al.:^41^

11 $$E_{\mathrm{MP}2}^{\infty }=\frac{E_{\mathrm{corr}}^{\mathrm{MP}2,X}X^{3}-E_{\mathrm{corr}}^{\mathrm{MP}2,Y}Y^{3}}{X^{3}-Y^{3}}$$

where *X* and *Y* represent basis sets; in this case, *X* = 3 for the cc-pVTZ basis set and *Y* = 4 for the cc-pvQZ basis set. Halkier et al. have reported that the values obtained with the TZ/QZ combination have a mean error of 1.3 kcal/mol and a maximum error of 3.25 kcal/mol for their sample calculations.

**Table 1:** HF, MP2, and CCSD­(T) Interaction Energies (kcal/mol) with an Approximate CBS (eqs 10 and 11) and DFT Interaction Energies with the aug-cc-pVTZ Basis Set for 32 Bimolecular Complexes^a^

system:		CCSD(T)-CBS	MP2-CBS	HF-CBS	ωB97XD	ωB97M-V	M06-2X-D	MN15	CAM-B3LYP-D
Mg(EDA)_2_(H_2_O)^2+^ with	catechol	–226.33	–226.04	–219.89	–224.91	–226.84	–229.79	–227.45	–226.94
	DNC	–203.36	–202.60	–193.28	–200.61	–203.06	–204.68	–201.98	–202.36
	Dopamine	–138.28	–138.07	–133.49	–137.64	–139.35	–141.93	–139.06	–139.83
	DOPAC	–377.87	–378.83	–359.52	–376.58	–378.27	–382.39	–378.47	–378.26
Zn(EDA)_2_(H_2_O)^2+^ with	catechol	–232.90	–233.27	–214.03	–223.75	–225.97	–229	–228.19	–224.71
	DNC	–202.95	–202.16	–184.59	–193.42	–195.12	–197.16	–196.15	–194.46
	dopamine	–137.14	–136.92	–124.28	–130.21	–130.98	–134.15	–132.88	–131.63
	DOPAC	–384.27	–385.04	–355.27	–373.01	–375.42	–378.12	–375.97	–373.78
methyl amine with	catechol	–10.10	–10.87	–4.44	–10.14	–10.02	–10.34	–9.23	–10.57
	DNC	–15.04	–15.94	–9.09	–15.45	–15.09	–15.84	–14.04	–16.07
	dopamine	–14.23	–15.15	–8.27	–14.39	–14.12	–14.49	–13.19	–14.86
	DOPAC	–10.51	–11.43	–3.86	–10.58	–10.29	–10.58	–9.59	–10.79
methanol with	catechol	–8.97	–9.54	–5.25	–8.99	–8.95	–9.21	–8.65	–9.55
	DNC	–11.86	–12.42	–8.94	–12.03	–12.03	–12.26	–11.51	–12.94
	dopamine	–12.79	–13.40	–9.34	–12.80	–12.77	–12.91	–12.27	–13.61
	DOPAC	–26.32	–27.26	–18.99	–26.27	–26.65	–27.71	–26.57	–27.45
benzene with	catechol	–7.35	–9.72	3.50	–7.01	–7.26	–8.05	–7.42	–6.26
	DNC	–8.71	–11.38	3.53	–7.74	–8.22	–8.39	–7.56	–7.21
	dopamine	–6.02	–8.85	7.36	–5.65	–5.73	–6.36	–6.13	–4.67
	DOPAC	–15.42	–18.17	–2.28	–14.80	–15.29	–16.04	–15.32	–14.33
indole with	catechol	–10.16	–12.26	–0.29	–10.18	–10.13	–10.76	–9.85	–9.28
	DNC	–13.71	–19.56	10.37	–10.75	–12.53	–13.52	–11.94	–9.73
	dopamine	–24.10	–26.23	–14.16	–24.70	–24.31	–24.84	–24.22	–23.77
	DOPAC	–18.78	–22.14	–0.64	–17.55	–18.24	–19.14	–18.01	–16.63
isobutane with	Catechol	–3.15	–3.94	2.82	–3.49	–3.08	–3.26	–3.47	–2.68
	DNC	–4.16	–4.93	2.64	–4.15	–4.00	–3.92	–3.98	–3.62
	dopamine	–4.42	–5.18	2.10	–4.92	–4.38	–4.52	–4.77	–4.02
	DOPAC	–5.21	–5.99	1.33	–5.54	–5.12	–5.23	–5.46	–4.65
methanethiol with	catechol	–7.61	–8.41	–1.28	–7.45	–7.45	–7.98	–7.8	–7.56
	DNC	–4.83	–5.65	1.79	–3.64	–4.43	–4.66	–4.52	–4.09
	dopamine	–15.72	–16.84	–7.25	–15.50	–15.40	–15.44	–15.2	–16.05
	DOPAC	–18.86	–20.62	–8.74	–18.19	–18.86	–19.73	–19.76	–18.83

a GD3 was used for M06-2X and GD3BJ was used for CAM-B3LYP.

CBS HF, MP2, and CCSD­(T) interaction energies for the complexes were found using the expression below with no counterpoise corrections applied:

12 $$E_{\mathrm{int}}=E_{\mathrm{AB}}-E_{A}-E_{B}$$

All MP2, CCSD, and CCSD­(T) calculations used a frozen core. Basis set superposition error (BSSE) is not accounted for in these CBS calculations, as the work by Miliordos and Xantheas shows that for binding energies of an electrostatically and dispersion-bound complex, non-BSSE corrected and BSSE-corrected calculations reach the same CBS limit.^42^ The LANL2DZ core potential was used for Zn only^43^ as the calculations for the Zn-complexes became intractable in terms of memory usage for integral transformations with larger basis sets. The loss of accuracy due to its use is discussed below.

Finally, four of the complexes from this work [complex (a) from each of the Figures 1–4 above, representing each of the four types of interactions included here] have been studied using the DLPNO-CCSD­(T) method.^44,45^ In this method, it is found that much of the electron correlation from each occupied orbital can be obtained from a nearby, or local, set of virtual orbitals. In particular, *pair-natural orbitals* have been successfully used to represent the virtual excitation space. Furthermore, these methods can be made to scale very well, including linearly, for relatively large molecular systems.^44,45^ These DLPNO-CCSD­(T) calculations use the aug-cc-pVTZ and aug-cc-pVQZ basis sets and include BSSE corrections in the same manner as the DFT calculations below.

### 2.3 Density Functional Theory Calculations

The interactions energies thirty-two complexes described above were calculated at the same geometries using twenty-one DFT methods: B97D3, ωB97XD, ωB97M-V, M06L, M06, M062X, M062X-D3, MN12SX, MN15, BLYP, B3LYP, BLYP-D3, CAM-B3LYP, CAM-B3LYP-D3, HCTH, τHCTHhyb, BMK, PBE, ωPBEhPBE, LC-ωHPBE, B2PLYPD3, and SVWN, all with the aug-cc-pVTZ^46,47^ basis set. As with the CCSD­(T) calculations, the LANL2DZ core potential was used for Zn only.^43^ The GD3 empirical dispersion correction was used for M06-2X as it is the only one available in the software; GD3 was also used for B2PLYPD3; the GD3 and GD3BJ corrections were used for both B3LYP and CAM-B3LYP, in order to compare the two. The energies were calculated with eq 12 with counterpoise corrections^48^ applied, meaning that in the calculation of each fragment molecule, the basis functions and DFT quadrature points from the opposite fragment were included. For most calculations, the standard SCF convergence procedure was successful, but in a few cases—notably some of counterpoise-corrected fragments calculated with the Minnesota functionals—the quadratic convergence procedure was needed. For both the Mg and Zn complexes with DOPAC, the counterpoise corrected fragment SCF for DOPAC did not converge for the M06L and MN12SX functionals, even with quadratic convergence, and so the interaction energies for those complexes with those functionals were calculated without counterpoise-correction and the average counterpoise-correction for the other Minnesota functionals was applied (+1 kcal/mol for the Mg complex, and +1.5 kcal/mol for the Zn complex).

### 2.4 DFT Basis Set Tests

Basis set convergence for the DFT methods was tested on a subset of eight of the complexes studied. Pitman et al. show that the aug-cc-pVTZ basis set used here is among the best-performing basis sets for DFT-based thermochemistry using three of the same functionals used here,^49^ but for the sake of completeness, further expansion of the basis set space was tested. Four hydrogen-bonded complexes (catechol and dinitrocatechol with methyl amine and methanol) and four π-stacking complexes (catechol and dinitrocatechol with benzene and indole) were chosen to represent systems where electrostatic, dipole–dipole interactions were dominant and where induction and dispersion interactions were dominant. Interaction energy calculations for each of the eight complexes were rerun with the aug-cc-pVQZ and def2-QZVPP basis sets to evaluate the effects of going from a triple-ζ basis set to a quadruple-ζ basis set. The comment by Gray et al. shows that the def2-QZVPD basis set is among the most accurate for DFT-based thermochemistry;^50^ the basis set used here differs from that one by substituting a second set of polarization functions for a set of diffuse functions.

### 2.5 DFT Optimization Tests

The same eight complexes used for the basis set tests were also used to test the effect of optimization with a DFT method on DFT-based energies, rather than using the same geometry for DFT calculations as that used for the CCSD­(T) calculations. This allows for the possibility that the DFT method may find a different minimum than the CCSD optimizations and that structure may yield a more “accurate” energy. The hydrogen-bonded complexes were optimized with CAM-B3LYP-D3/aug-cc-pvtz starting from the CCSD-optimized geometries in order to find the same relative minima. The π-stacking complexes were optimized with M062X-D3/aug-cc-pVTZ starting from the CCSD-optimized geometries in order to find the same relative minima as well. Interaction energies were then computed with the two DFT methods and aug-cc-pVTZ. Wang et al. have shown that geometries of reaction complexes are relatively insensitive to basis set choice, and that the triple-ζ set used here is within the range of stable basis sets tested (meaning that resulting energies do not vary greatly).^51^ They do suggest that the basis set effect may be greater for metal-containing complexes, such as the Zn-bearing complexes they tested, which are similar to the Zn-based complexes studied here. Even so, the resulting energies from the metal-based complexes differed by only ∼1.5 kcal/mol between basis sets.

## 3 Results

All of the CCSD­(T), MP2, and HF CBS results are reported in Table 1, along with a selection of DFT results. Summaries of the DFT results are reported in Table 2. Full results including structural parameters are reported in the Supporting Information. Noncounterpoise-corrected CCSD­(T)/cc-pVDZ and MP2/cc-pVQZ results are reported in Table S5 in the Supporting Information. While the CCSD­(T)/cc-pVDZ energies are not accurate compared to CCSD­(T)/CBS, the MP2/cc-pVQZ energies are much closer in value to the MP2/CBS values.

**Table 2:** Average Absolute Difference (AAD) for Each DFT Method Compared to CCSD for Ionic, h-Bond, π-Stacking, and Other Interactions (kcal/mol)^a^

method	ionic	H-bond	π-stack	other	total
B97D3	13.58	0.65	0.77	0.41	3.85
ωB97XD	5.38	0.12	0.89	0.43	1.70
ωB97M-V	4.01	0.12	0.37	0.16	1.17
M06L	5.68	0.74	2.46	1.03	2.48
M06	6.65	0.88	2.89	1.22	2.91
M062X	3.99	0.26	0.54	0.63	1.36
M062X-GD3	3.97	0.44	0.48	0.27	1.29
MN12SX	7.34	1.92	3.00	1.79	3.51
MN15	3.50	0.66	0.55	0.38	1.27
BLYP	26.76	3.80	13.75	6.69	12.75
B3LYP	18.41	2.64	11.54	5.59	9.54
B3LYP-GD3BJ	6.57	0.36	0.72	0.35	2.00
CAM-B3LYP	11.45	1.05	8.70	4.09	6.32
CAM-B3LYP-GD3BJ	4.53	0.75	1.55	0.39	1.81
PBE	20.19	1.36	8.78	3.80	8.53
wPBEhPBE	19.22	1.17	8.54	3.71	8.16
LC-wHPBE	11.66	2.14	8.14	4.21	6.54
HCTH	29.63	4.15	13.52	5.67	13.24
tHCTHhyb	16.77	1.88	9.33	4.43	8.10
BMK	9.23	1.92	6.53	3.76	5.36
SVWN	3.49	5.81	1.51	1.74	3.14
B3PLYPD3	11.52	1.73	6.70	3.21	5.79
HF-CBS	14.86	5.21	13.96	7.17	10.30
MP2-CBS	0.55	0.77	3.01	0.95	1.32
CCSD(T)-CBS	0.00	0.00	0.00	0.00	0.00

a All calculations use the aug-cc-pVTZ basis set except where noted.

### 3.1 Ionic/electrostatic Complexes

Each of the metal-ion complexes (Figure 1) was optimized with MP2/cc-pVDZ. The metal-catechol distances (as measured by the Mg^2+^···O^–^ distance) are reported in the Supporting Information, Table S7. The ligand-Mg^2+^ MP2 distances ranged from 1.98 to 2.02 Å, and the ligand-Zn^2+^ MP2 distances ranged from 2.00 to 2.04 Å. Harrison et al. obtained crystal structures for catecholic ligands bound to the COMT enzyme and found ligand-Mg^2+^ distances of 2.1 and 2.2 Å.^52^ Thus, the distances studied here are slightly shorter than those from a crystal structure, though this difference is likely attributable to crystallization effects or solvent effects. CCSD­(T) and MP2 CBS interaction energies for Mg^2+^ and Zn^2+^ with the four catechols (Table 1) were very similar between the two metal ions, with the negatively charged DOPAC having the strongest interactions with the compounds and the positively charged dopamine having the weakest interactions. The two neutral catechols had similar interaction energies with the metal complexes, with catechol having stronger interactions than dinitrocatechol by 23 and 30 kcal/mol for Mg^2+^ and Zn^2+^, respectively. The CCSD­(T) and MP2 energies for all eight complexes were in close agreement, with MP2 within 1 kcal/mol of the CCSD­(T) values in all cases. HF interaction energies were all within 3 and 5% of the CCSD­(T) values. Of the twenty-one DFT methods studied here, the smallest absolute average difference between the DFT interaction energy and the CCSD­(T)/CBS interaction energy was 3.50 kcal/mol, for the MN15 functional. Both M062X and M062X-D had similar differences of 3.99 and 3.97 kcal/mol, ωB97M-V had a slightly larger difference of 4.01, while CAM-B3LYP-D3 was the fifth most accurate with a difference of 4.53 kcal/mol. The rather large differences between the DFT energies and the CCSD­(T) energies are due largely to the Zn^2+^-complexes. If the average absolute differences for the DFT methods are calculated using only the Mg^2+^-based complexes, then three functionals agree with CCSD­(T) within less than 1 kcal/mol: ωB97M-V, CAM-B3LYP-D3, and MN15 with accuracies of 0.58, 0.89, and 0.98 kcal/mol. These are followed by ωB97XD and M06L with accuracies of 1.54 and 1.57 kcal/mol. This larger error may be due to the use of a core potential for Zn^2+^ in these calculations. Despite this increased error, these calculations were included in this work as many calculations on metal complexes by practitioners are carried out using core-potentials, and so it should be noted that their use can affect the accuracy of results by 2.5 to 3 kcal/mol in systems such as those studied here. In all cases, both BLYP and B3LYP are in error by 13 or more kcal/mol, showing that both range-separated exchange and empirical dispersion are needed to make these functionals accurate. Both the PBE-based functional series and the HCTH-based functional series have large differences in all cases, and the inclusion of range-separated or hybrid exchange does not raise the accuracy to a good level.

### 3.2 Hydrogen-Bonded Complexes

Figure 2 shows the hydrogen-bonded complexes studied here, which were each optimized with CCSD/cc-pVDZ. These compounds can be characterized by the hydrogen-bond length; for the first four complexes, this bond length is between the hydroxyl group proton of the catechol and the nitrogen on the methylamine. All values are reported in the Supporting Information, Table S7. Lu et al. obtained crystal structures of the SULT enzyme bound to dopamine, and found catechol—amine hydrogen-bond lengths between 1.7 and 1.8 Å.^53^ The CCSD-optimized structures in Table S7 range between 1.64 and 1.90 Å, placing the experimental structures squarely in between the calculated distances. The CCSD­(T)/CBS interaction energies ranged between −10 and −15 kcal/mol for these complexes, with the MP2/CBS interaction energies all within 1 kcal/mol of the CCSD­(T) values. HF/CBS values were considerably lower, ranging between −4 and −9 kcal/mol. The next four hydrogen-bonded complexes were analogues of the four above with methanol replacing the methylamine. These complexes are characterized by the catechol-hydroxyl to methanol-O OH···O distances, which can be found in the Supporting Information, Table S7. The crystal structures of Elandson et al. show catechol-hydroxyl bond lengths of ∼1.5 to 1.8 Å,^54^ compared to the 1.66 to 1.83 Å in Table S7. Thus, the CCSD distances are biologically relevant. The DOPAC-methanol complex had an interesting structure, with the hydroxyl group on methanol forming two hydrogen bonds: one with the intended phenolic hydroxyl group on DOPAC, and one with the charged carboxyl group on DOPAC. The CCSD­(T) CBS interaction energies for the first three complexes were similar to those for the methyl amine complexes (between −9 and −13 kcal/mol), but the DOPAC complex was much stronger, −26 kcal/mol/due to the additional ion/dipole bond present. Again, the MP2/CBS interaction energies were within 1 kcal/mol of CCSD­(T), and the HF/CBS energies were considerably less attractive. While HF does not generally model hydrogen-bonding as well as MP2 or CCSD­(T), it can be more accurate than what is shown here. Riley et al. showed that, with an aug-cc-pVTZ basis set, MP2 had an error of 0.3 kcal/mol against a database of ten hydrogen-bound systems of biological relevance, while HF had an error of 1.73 kcal/mol.^55^ That MP2 error is comparable to that found in this work (AAD of 0.77 kcal/mol), but the HF error is smaller than that found in this work (5.21 kcal/mol). The HF/CBS energies’ inability to correctly model the hydrogen-bond energies here is due to the fact that the proton donor in all of these complexes is the hydroxyl group of a substituted phenol, and so induction effects play a large role in the partial charge of the donated proton. MP2 and CCSD­(T) can model this induction effect, but HF cannot. Of the twenty-one DFT methods studied here (Table 2), nine had interaction energies within 1 kcal/mol of the CCSD­(T)/CBS energies. The functionals which produced the energies closest to CCSD­(T) were ωB97XD, ωB97M-V, M062X, B3LYP-GD3BJ, and M062X-GD3, in that order. Of the other five with subkcal/mol accuracy, one included empirical dispersion, and one was a range-separated hybrid that also included empirical dispersion. All eight were either meta-GGA or a range-separated GGA, or included empirical dispersion. Clearly, some nonlocality beyond the GGA or global hybrid level was needed to match the CCSD­(T) interaction energies for these catechol benchmark hydrogen-bonded systems.

### 3.3 π-Stacking Complexes

Eight complexes were used to analyze the π-stacking structure optimization and complexation energy performance of the twenty-one DFT methods studied here (Figure 3). π-stacked complexes can take on three conformations: stacked sandwich, slipped sandwich, and T-shaped. The catechol-benzene complex optimized to a T-shaped conformation with CCSD (Figure 5b). But as the weaker, sandwich complex was desired for this analysis, the MP2 slipped-sandwich structure (Figure 5a) was used. These sandwich complexes can be seen in crystal structures such as the aromatic ligand bound to MAOB by Morgan and Hurley (ring–ring distance of 3.7 Å),^56^ and, as they are slightly weaker than the T-shaped complexes, they serve as a more rigorous test of the DFT methods studied here. The dinitrocatechol-benzene complex optimized to a slipped sandwich conformation, owing to the extended π-system from the nitro-substituents. The nitro substituent, being strongly electron-withdrawing, also acts inductively, polarizing the π-system and allowing stronger interactions compared to the catechol complexes. The dopamine-benzene complex again had two different structures found by optimization. The MP2 calculation optimized to a slipped sandwich conformation (Figure 5c), while the CCSD calculation found a cation-π structure (Figure 5d). For the following analysis, the MP2-optimized slipped sandwich conformation was used. Finally, the DOPAC-benzene complex optimized to a slipped sandwich structure wherein the carboxyl group from the DOPAC formed a weak interaction with the positive regions on the H nuclei around the benzene ring. The next four complexes studied were the indole analogues of the benzene complexes studied above (Figure 3). The first three complexes all optimized to slipped sandwich conformations, though in the case of the dopamine-indole complex, the charged −NH_3_^+^ group did interact with the indole as well, increasing the strength of the interaction. The final π-stacking complex studied here was DOPAC-indole. This complex optimized to a slipped sandwich conformation with the anionic carboxyl group interacting with the more positive region of the indole double-ring, furthest away from the N atom (Figure 5).

**Figure 5:**
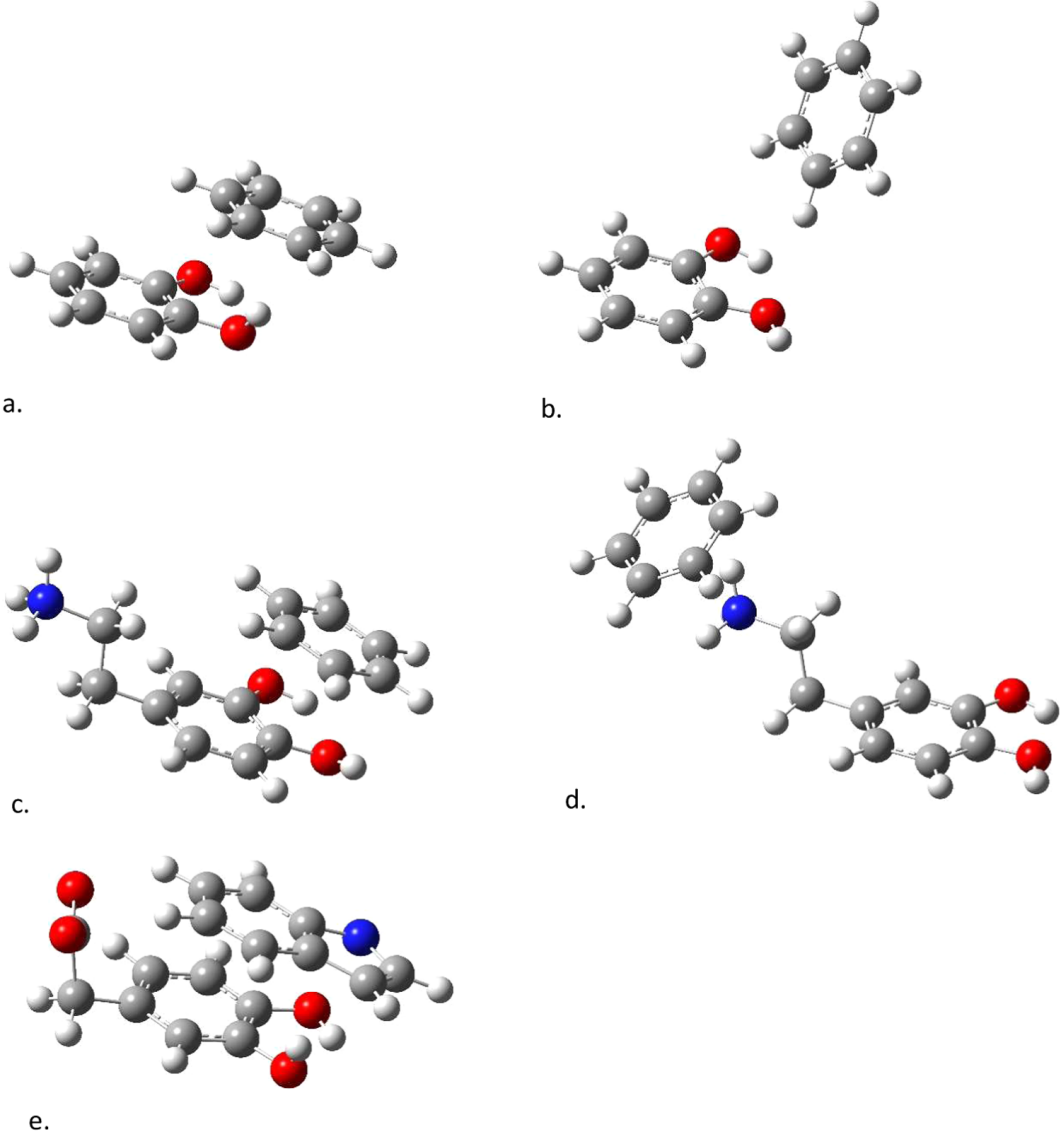
Optimized structures (cc-pVDZ basis set) for (a) catechol-benzene with MP2, (b) catechol-benzene with CCSD, (c) dopamine-benzene with MP2, (d) dopamine-benzene with CCSD, (e) DOPAC-indole with CCSD.

The CCSD­(T)/CBS interaction energies for the catechol-benzene complexes ranged from about −6 kcal/mol (dopamine) to about −14 kcal/mol (DOPAC). The catechol-indole complexes all had stronger energies, as would be expected due to the larger π-system. This is due to the fact that the π-stacking interactions are dominated by dispersion and induction forces, which in turn are proportional to the polarizabilities, α, of the molecules. Work by Zhang et al. shows that the ωB97xD/aug-cc-pVTZ model chemistry predicts an isotropic polarizability of 12.1 Å^3^ for benzene and 18.6 Å^3^ for indole.^57^ The ratio of the polarizabilities is 0.53, which is in line with the ratios for the catechol-benzene/catechol-indole interaction energies (0.38) and the DNC-benzene/DNC-indole interaction energies (0.57). The dopamine-indole complex has the strongest attraction, at −24 kcal/mol, as the positive amine bonded to the lone pair on the indole’s N. While the metal-complexes and hydrogen-bonded complexes showed good agreement between CCSD­(T)/CBS and MP2/CBS, for the π-stacking complexes, MP2 overestimates the attraction between the molecules by between −2 and −6 kcal/mol. The π-stacking is a more subtle interaction than the ion-dipole interactions in the metal complexes or the hydrogen bonds, so MP2 is expected to overestimate the energy. HF/CBS predicts repulsive interactions for three of the four benzene interactions and underestimates all of the indole interactions. Of the twenty-one DFT methods studied here, six of them agreed with CCSD­(T)/CBS within 1 kcal/mol. In order, starting with the most accurate, they were: ωB97M-V, M062X-D3, M062X, MN15, B3LYP-GD3BJ, B97D3, and ωB97XD. Again, nonlocality beyond the GGA or hybrid level is needed, as these five are either meta-GGA or include empirical dispersion. HCTH and BLYP performed about as poorly as HF, and B3LYP also had very poor performance, though CAM-B3LYP-D3 had accuracy within 1.5 kcal/mol of CCSD­(T)/CBS, showing that the poor performance of BLYP could be systematically improved. Adding exact exchange and kinetic energy density to HCTH, however, did not improve its performance significantly.

### 3.4 Other Complexes

The next four complexes studied consisted of the four catechols in complex with isobutane (Figure 4). In all cases, the catechols formed a complex with the isobutane stacked above the phenyl ring (away from the hydroxyl groups) at a distance of between 3.5 and 4.0 Å. This nonpolar, dispersion-dominated interaction models how catechol-based molecules bind to nonpolar amino-acid residues, including leucine, isoleucine, valine, glycine, and alanine. The crystal structure of ALDH bound to an aromatic ligand by Morgan and Hurley shows a distance from the ligand to an isoleucine of 3.7 Å,^56^ squarely in the range found here. The CCSD­(T)/CBS interaction energies were between ∼−3 and ∼−5 kcal/mol for the complexes, with the neutral catechol and dinitrocatechol complexes being slightly weaker than the charged complexes. The MP2/CBS interaction energies were slightly stronger (more negative) than the CCSD­(T) energies, but the difference was less than 1 kcal/mol in all cases. HF/CBS found positive (repulsive) interaction energies in all cases, as would be expected.

The final four complexes studied here are dipole-π systems with methanethiol complexed to the four catechols (Figure 4e–h). These interactions are representative of interactions between catecholic ligands and amino acid residues like cysteine and methionine, as well as asparagine and glutamine. The catechol complex optimized to a dipole–dipole dominated system with the catechol hydroxyl group donating a proton to the sulfur atom on the thiol (Figure 6a) with an OH···S distance of 2.35 Å. The dinitrocatechol-methanethiol complex formed an intramolecular hydrogen bond between the hydroxyl group and the nitro group, and the electron density on the sulfur nucleus bonded to the positive region in the middle of the intramolecular hydrogen bond (Figure 6b). The dopamine-methanethiol complex formed an ion-dipole interaction between the −NH_3_^+^ group and the sulfur atom (Figure 6c), with the NH···SH distance of 2.25. The DOPAC-methanethiol complex formed an ion-dipole bond between an oxygen atom on the carboxyl group and the proton from the thiol. There was also a dipole–dipole interaction between a hydroxyl group on DOPAC and the S atom (Figure 6d). The crystal structure of MAOB bound to a phenolic ligand of Binda et al. shows a catecholic-hydroxyl-thiol hydrogen bond distance of about 2.0 Å, which is shorter than the bond lengths reported here.^58^ This may be attributable to the fact that the experimental structure is phenolic rather than catecholic.

**Figure 6:**
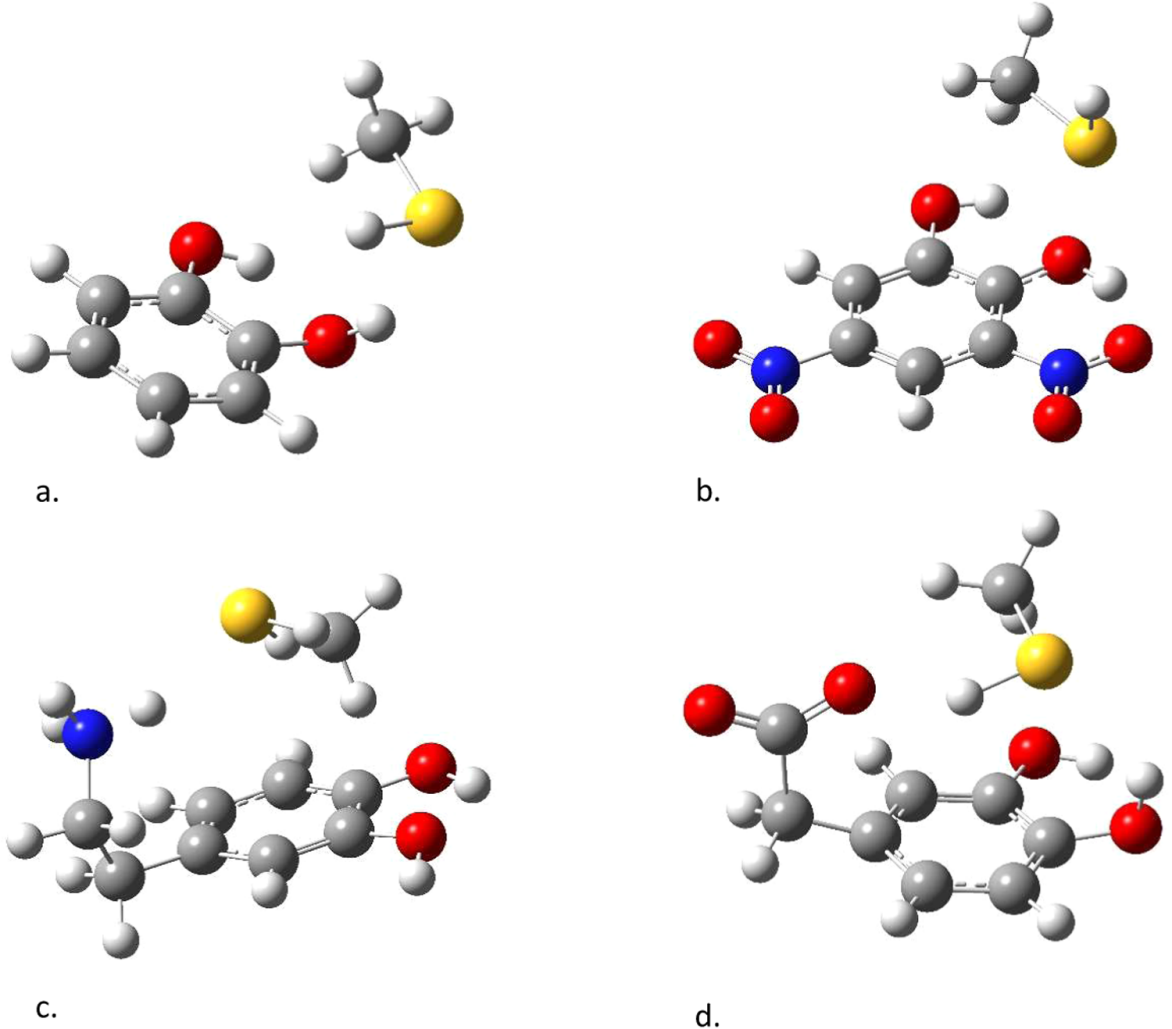
CCSD/cc-pVDZ optimized structures of (a) catechol-methanethiol, (b) dinitrocatechol-methanethiol, (c) dopamine-methanethiol, (d) DOPAC-methanethiol.

The CCSD­(T)/CBS interaction energies were ∼−7 and ∼−19 kcal/mol, with the dinitrocatechol complex being the weakest, as the intramolecular hydrogen bond in dinitrocatechol weakens the interaction with the thiol group. MP2/CBS overestimated the strength of the attraction by 1–2 kcal/mol, and HF/CBS underestimated the attraction by about half for all complexes except the dinitrocatechol complex, for which it found a positive/repulsive interaction. Of the DFT methods studied here for these eight complexes, ωB97M-V had the closest values to CCSD­(T)/CBS with an average difference of 0.16; this was followed by M062X-D3, B3LYP-GD3BJ, and MN15. CAM-B3LYP-GD3BJ was a close fifth, with both B97D3 and ωB97XD following and having almost identical accuracy. Overall, 7 DFT methods were within 1 kcal/mol of CCSD­(T) and an additional 4 were within 2 kcal/mol of CCSD­(T). It is clear that having empirical dispersion is necessary for a DFT method to achieve sub-kcal/mol accuracy for these complexes, other than the MN15 method, which performed well regardless. Both HCTH and τHCTHhyb performed poorly, showing that for these functionals, inclusion of kinetic energy density and exact exchange does not improve the performance in an appreciable way. Similarly, while BLYP, B3LYP, and CAM-B3LYP performed poorly, B3LYP-GD3BJ and CAM-B3LYP-GD3BJ performed very well, suggesting that it is the empirical dispersion that had the greatest effect.

### 3.5 DFT Basis Set Tests

Interaction energy values for the eight complexes with three basis sets are shown in Table 3. For each of the four hydrogen-bonded complexes, the differences in the energies between the aug-cc-pVTZ basis set used in the DFT benchmarking above and the quadruple-ζ basis sets tested are minimal. Triple zeta/quadruple-ζ differences were between 0.01 and 0.06 kcal/mol, all of which are right at or below the threshold of accuracy for DFT methods. The π-stacking complexes showed slightly larger differences, up to 0.18 kcal/mol, which is still close to the threshold for DFT accuracy, and so may be counted as negligible. For the hydrogen-bonded complexes, interaction energies became slightly more attractive with quadruple-ζ basis sets, while the interaction energies for the π-stacking complexes became slightly less attractive with the quadruple-ζ basis sets. Overall, the aug-cc-pVTZ basis set is established as sufficiently large to describe the DFT-based interaction energies.

**Table 3:** Basis Set Convergence Behavior for Interaction Energies for Eight Complexes with the Same Geometries Used for CCSD­(T)/CBS Calculations^a^

complex	functional	basis set	IE
methyl amine/catechol	CAM-B3LYP-D3BJ	aug-cc-pVTZ	–10.57
		aug-cc-pVQZ	–10.58
		def2-QZVPP	–10.56
methyl amine/DNC	CAM-B3LYP-D3BJ	aug-cc-pVTZ	–16.07
		aug-cc-pVQZ	–16.08
		def2-QZVPP	–16.07
methanol/catechol	CAM-B3LYP-D3BJ	aug-cc-pVTZ	–9.55
		aug-cc-pVQZ	–9.59
		def2-QZVPP	–9.6
methanol/DNC	CAM-B3LYP-D3BJ	aug-cc-pVTZ	–12.94
		aug-cc-pVQZ	–12.98
		def2-QZVPP	–13
benzene/catechol	M06-2X-D3BJ	aug-cc-pVTZ	–8.05
		aug-cc-pVQZ	–7.97
		def2-QZVPP	–7.92
benzene/DNC	M06-2X-D3BJ	aug-cc-pVTZ	–8.39
		aug-cc-pVQZ	–8.37
		def2-QZVPP	–8.29
indole/catechol	M06-2X-D3BJ	aug-cc-pVTZ	–10.76
		aug-cc-pVQZ	–10.64
		def2-QZVPP	–10.58
indole/DNC	M06-2X-D3BJ	aug-cc-pVTZ	–13.52
		aug-cc-pVQZ	–13.51
		def2-QZVPP	–13.39

a All values in kcal/mol.

### 3.6 DFT Optimization Tests

Table 4 shows the results of using two different geometry optimizations on DFT-based interaction energies. Each of the eight complexes chosen for this study was optimized with CCSD/cc-pvDZ and with the DFT method in question and the aug-cc-pVTZ basis set. In all but one case, it can be seen that the optimization with DFT leads to a slightly stronger interaction. The difference in interaction energy between the two optimization methods is 0.39 kcal/mol on average for hydrogen-bonded complexes, and 0.43 kcal/mol for the π-stacking complexes. It should be noted that for the π-stacking complexes, the two complexes with benzene had very small differences (average of 0.09 kcal/mol), while the two complexes with indole had larger differences (average of 0.77 kcal/mol). Overall, it is shown that the DFT-based optimization can lead to slightly stronger DFT-based interaction energies, as each computational method will find its own, slightly different, unique minimum, yielding a method-specific interaction energy. The differences are small, though, in these cases.

**Table 4:** Interaction Energies for CCSD/cc-pvdz^b^ Optimized and DFT/aug-cc-pvdz Optimized Geometries for Eight Complexes^a^

complex	energy method	optimization method	IE
methyl amine/catechol	CAM-B3LYP-D3/aug-cc-pVTZ	CAM-B3LYP-D3/aug-cc-pVTZ	–11.21
		CCSD/cc-pvdz	–10.57
methyl amine/DNC	CAM-B3LYP-D3/aug-cc-pVTZ	CAM-B3LYP-D3/aug-cc-pVTZ	–16.81
		CCSD/cc-pvdz	–16.07
methanol/catechol	CAM-B3LYP-D3/aug-cc-pVTZ	CAM-B3LYP-D3/aug-cc-pVTZ	–9.74
		CCSD/cc-pvdz	–9.55
methanol/DNC	CAM-B3LYP-D3/aug-cc-pVTZ	CAM-B3LYP-D3/aug-cc-pVTZ	–13.3
		CCSD/cc-pvdz	–12.94
benzene/catechol	M06-2X-D3/aug-cc-pVTZ	M06-2X-D3/aug-cc-pVTZ	–7.99
		MP2/cc-pvdz	–8.05
benzene/DNC	M06-2X-D3/aug-cc-pVTZ	M06-2X-D3/aug-cc-pVTZ	–8.5
		CCSD/cc-pvdz	–8.39
indole/catechol	M06-2X-D3/aug-cc-pVTZ	M06-2X-D3/aug-cc-pVTZ	–11.71
		CCSD/cc-pvdz	–10.76
indole/DNC	M06-2X-D3/aug-cc-pVTZ	M06-2X-D3/aug-cc-pVTZ	–14.1
		CCSD/cc-pvdz	–13.52

a The GD3BJ correction was used for both M06-2X and CAM-B3LYP. All values in kcal/mol.

b MP2/cc-pvdz in one case, as indicated.

### 3.7 Empirical Dispersion Comparison

Both the B3LYP and CAM-B3LYP functionals were tested with GD3 and GD3BJ empirical dispersion corrections (Table 5). For the CAM-B3LYP functional, it can be seen that the overall accuracy of the two dispersion corrections is nearly identical, but the GD3 correction performs slightly better on ionic/electrostatic interactions, and GD3BJ performs better on hydrogen-bond/electrostatic interactions. Performance in the other two categories is very similar between the two correction methods. For B3LYP, the GD3BJ correction is more accurate overall by a modest amount (0.14 kcal/mol). For the predominantly ionic/metal interactions, the two corrections are nearly identical, while GD3BJ is more accurate for hydrogen bonding (which includes induction and dispersion) and the induction and dispersion dominated π-stacking interactions. GD3 is nominally more accurate for the “other” interactions, which include dispersion and weak-hydrogen bonding. Overall, either could be used to good effect.

**Table 5:** Average Absolute Difference (AAD) for B3LYP and CAM-B3LYP with GD3 and GD3BJ Empirical Dispersion Corrections Compared to CCSD for Ionic, H-Bond, π-Stacking, and Other Interactions (kcal/mol)^a^

method	ionic/metal	h-bond	π-stack	other	total
B3LYP-D3	6.54	0.59	1.14	0.27	2.14
B3LYP-D3BJ	6.57	0.36	0.72	0.35	2.00
CAM-B3LYP-D3	4.18	1.21	1.51	0.35	1.81
CAM-B3LYP-D3BJ	4.53	0.75	1.55	0.39	1.80

a All calculations use the aug-cc-pVTZ basis set except where noted.

### 3.8 DLPNO-CCSD­(T) Calculations

Table 6 shows a comparison between DLPNO-CCSD­(T) with two different basis sets and the CCSD­(T)/CBS methods reported above. These comparisons are performed for four of the compounds studied here, one of each interaction type. The DLPNO-CCSD­(T) calculations include BSSE corrections are also extrapolated to the CBS limit using eq 11 in two ways: first using a double/triple-ζ extrapolation [CBS­(D,T)] and then using a triple/quadruple-ζ extrapolation [CBS­(T,Q)]. While the DLPNO-CCSD­(T) energies with the aug-cc-pVTZ and aug-cc-pVQZ basis sets are well above the CCSD­(T)/CBS energies and have not converged with basis set, the extrapolated DLPNO-CCSD­(T)/CBS energies come within at least 97% of the CCSD­(T)/CBS energy, with some as close as 1% from the CCSD­(T)/CBS energy. While DLPNO-CCSD­(T) is a faster method than conventional CCSD­(T), the size of basis set required to obtain an energy with the same accuracy as the more accurate DFT methods reported here requires considerably more compute than DFT, and so, while it may be used as a benchmark or standard, it is not a likely candidate for routine use.

**Table 6:** Interaction Energies between Catechol and Four Other Molecules Using Either the Complete-Basis Set CCSD­(T) Extrapolation Described Above, or DPLNO-CCSD­(T) with Two Different Basis Sets, aug-cc-pVTZ (augTZ) and aug-cc-pVQZ (augQZ, kcal/mol)^a^

catechol w/	DPLNO/aug,DZ	DPLNO/aug,TZ	DPLNO/aug,QZ	DPLNO/CBS(D,T)	DPLNO/CBS(T,Q)	CCSD(T)/CBS
Mg(EDA)2(H2O)2+	–222.59	–225.40		–226.58		–226.33
methyl amine	–8.61	–9.58	–9.84	–9.99	–10.02	–10.10
benzene	–6.02	–7.09	–7.27	–7.54	–7.40	–7.35
isobutane	–2.59	–2.93	–3.00	–3.07	–3.05	–3.15

a Extrapolated DLPNO-CCSD­(T)/CBS energies are also reported.

## 4 Discussion and Conclusions

The MP2/CBS calculations overestimate the CCSD­(T)/CBS interaction energies for all complexes, but the difference is most pronounced for the weaker, π-stacking and other induction and dispersion-based interactions. This phenomenon is well-known, and has been demonstrated by Tsuzuki et al. for toluene dimers^59^ where the MP2/CBS interaction energies can be more than double the CCSD­(T)/CBS energies. Sinnokrot and Sherrill showed that, for benzene dimers, MP2 with a large basis overestimates CCSD­(T)/CBS by between 0.75 and 2 kcal/mol.^60^ The current work shows that MP2/CBS overestimates CCSD­(T) by at least 2 and as much as 4 kcal/mol for the π-stacking complexes studied here.

The functionals with the best accuracy compared to the CCSD­(T)/CBS across all interaction types were ωB97M-V, M062X-D3, MN15, M062X, ωB97XD, CAM-B3LYP-GD3BJ, and B3LYP-GD3BJ. Estimates from the literature show that the small-basis set correction term used for the CBS extrapolation could be ± 0.25 kcal/mol.^40^ The most accurate functional found here has an error compared to CCSD­(T)/CBS of 1.17 kcal/mol across all complexes, so, including the potential error in the extrapolation, ωB97M-V would still be within 1.42 kcal/mol of CCSD­(T)/CBS. The high ranking of CAM-B3LYP-GD3BJ, M062X-D3, and ωB97XD across all interaction types studied here suggests that the dispersion correction can be crucial for a consistent description of all interactions needed when studying protein–ligand binding with DFT (Table 2), although the nonlocal correlation from ωB97M-V is arguably more capable of obtaining good accuracy. This work shows that the choice of GD3 and GD3BJ leads to slight differences in accuracy when paired with B3LYP and CAM-B3LYP. GD3BJ has overall greater accuracy, but GD3 can be more accurate for ionic interactions and pure dispersion interactions. The inclusion of both M062X and MN15 in the top five rankings for all interaction types implies that hybrid, meta-GGA methods can also perform well. Finally, the presence of ωB97M-V, ωB97XD, and CAM-B3LYP-D3 in the top five methods suggests that range-separation can help a functional achieve high accuracy. Nonhybrid GGA methods and methods based on PBE and HCTH performed poorly in all cases.

Some of the current work agrees with the trends found in the earlier work of Boese,^61^ wherein databases of hydrogen-bonded complexes were tested with a suite of DFT methods, with and without empirical dispersion corrections. When comparing average errors from the set of 16 hydrogen bonds in that work with the average errors from the eight hydrogen bonds in this work, several points of agreement can be found. Both the current work and that work found that the range-separated MN12SX functional performed worse for hydrogen bonds than the M06 functionals. Boese often finds that the “pure” functionals perform more accurately than those with empirical dispersion corrections. While that trend may hold for hydrogen bonds where electrostatic interactions are the primary contributor, we have shown that induction and dispersion effects can play a large role in hydrogen bonds, such as in the comparison between HF and MP2 and CCSD­(T) hydrogen bond energies in this work. The current work shows that empirical dispersion decreases the accuracy of M062X slightly, but increases the accuracy of CAM-B3LYP, so no clear conclusions can be drawn.

Liao et al.^62^ also studied the accuracy of DFT methods with empirical dispersion corrections, but to examine complexes of O_2_ and CO_2_ binding to a model for myoglobin containing a porphyrin ring and the five closest amino acid residues. They found that the D3 correction on the BP,^20,63^ revPBE,^24^ and B3LYP functionals produced poor results for structure and binding energy compared with experimental results. The closest analogous complexes in the current work are the catechol-metal complexes. For these systems, this work finds that dispersion corrections to M062X have a negligible effect, while the dispersion correction reduces the error of CAM-B3LYP by over 50%. Ehrlich et al.,^38^ however, found that DFT with empirical dispersion (particularly TPSS-D and B2PLYP-D) reproduced high-level *ab initio* results for π-stacking interactions accurately, while MP2 could be in error by 200–300%. The current work also finds several dispersion-corrected functionals that agree with the CCSD­(T)/CBS π-stacking energies to within 1 kcal/mol (B97D3 ωB97XD and M062X-D3), while MP2 was in error by about 40%. Again, this shows that dispersion can have large effects on electrostatic interactions such as hydrogen bonds and metal coordination.

For more general comparisons, Kang and Byun studied several of the same methods examined here (ωB97XD, M062X, LC-ωHPBE, and B3LYP) for their performance in replicating the structures and energetics of small peptides.^64^ The authors used large-basis set MP2 calculations as the comparison standard, whereas the current work uses CCSD­(T) as a standard, with an augmented, triple-ζ basis set for the DFT calculations. Contrary to the current results, they found ωB97XD to be the best performer of these methods, followed by M062X, LC-ωPBE, and then B3LYP. Using the overall average absolute difference metric in this work, this work found that M062X outperforms ωB97XD, though the relative rankings of LC-ωHPBE and B3LYP agree with that work. The structures used by Kang and Byun are dependent on internal torsion angles and the calculations were done with implicit solvent, so they are not directly comparable to this work, which depends on longer-range nonbonded interactions and are done in the gas phase. Furthermore, MP2 does often produce larger magnitude complexation energies compared to CCSD, and so some of the agreement found by these authors could be attributable to the nonvariational energy of MP2.

In an interesting note, in 2006, Siegbahn studied the use of DFT for metal complexes in enzymes and stated that it would be unlikely that better quantum chemical methods than B3LYP would be developed in the “near future” for simulating coordination to metal centers.^65^ In this work, it is found that the M06 family of functionals (first published in the same year as Siegbahn’s assessment), ωB97M-V, ωB97XD, and CAM-B3LYP-D3 all exceed the accuracy of B3LYP for metal complexation by far.

Finally, the DPLNO-CCSD­(T) method, extrapolated to the CBS limit from double/triple-ζ or triple/quadruple-ζ comes within 3–1% of the CCSD­(T)/CBS energies calculated here. This method is considerably faster than CCSD­(T) but is still much slower than DFT methods with similar accuracy.

## Supplementary Material



## Notes

Complexation energies for complexes of four catechols (catechol, dinitrocatechol, dopamine, and DOPAC) with eight counter-molecules: Mg­(EDA)_2_(H2O)_2_+, Zn­(EDA)_2_(H2O)_2_+, methyl amine, methanol, benzene, indole, isobutane, and methanethiol (XLSX)
